# Protected Areas in South Asia Have Not Prevented Habitat Loss: A Study Using Historical Models of Land-Use Change

**DOI:** 10.1371/journal.pone.0065298

**Published:** 2013-05-31

**Authors:** Natalie E. Clark, Elizabeth H. Boakes, Philip J. K. McGowan, Georgina M. Mace, Richard A. Fuller

**Affiliations:** 1 Centre for Agri-Environmental Research, School of Agriculture, Policy and Development, University of Reading, Reading, Berkshire, United Kingdom; 2 Centre for Population Biology, Imperial College London, Ascot, Berkshire, United Kingdom; 3 Centre for Biodiversity and Environment Research, Department of Genetics, Evolution and Environment, University College London, London, United Kingdom; 4 School of Biology, Newcastle University, Newcastle upon Tyne, United Kingdom; 5 School of Biological Sciences, University of Queensland, Brisbane, Queensland, Australia; University of Copenhagen, Denmark

## Abstract

Habitat loss imperils species both locally and globally, so protection of intact habitat is critical for slowing the rate of biodiversity decline. Globally, more than 150,000 protected areas have been designated with a goal of protecting species and ecosystems, but whether they can continue to achieve this goal as human impacts escalate is unknown. Here we show that in South Asia, one of the world's major growth epicentres, the trajectory of habitat conversion rates inside protected areas is indistinguishable from that on unprotected lands, and habitat conversion rates do not decline following gazettement of a protected area. Moreover, a quarter of the land inside South Asia's protected areas is now classified as human modified. If the global community is to make significant progress towards the Convention on Biological Diversity's Aichi Target on protected areas, there is an urgent need both to substantially enhance management of these protected areas and to develop systematic conservation outside the formal protected area system.

## Introduction

Since the last ice age, around 75% of the natural vegetation across the planet has been cleared or otherwise dominated by human activity [1], [2]. This has precipitated a global biodiversity crisis in which rates of species extinction far exceed background predictions [3], [4], [5], [6], [7], and the consequent impacts upon human well-being are becoming apparent [8]. The loss of natural habitat is widely forecasted to continue with a further 10 million km^2^ of natural habitat predicted to be converted for agriculture by 2050 [9] and many biodiversity hotspots decreasing by more than 50% over the next century [10]. One of the major global responses to this rapid habitat loss has been the inception and growth of a protected area network, consisting of more than 150,000 individual sites [11]. Now occupying 12.7% of the Earth's land surface [12], protected areas are generally considered effective at abating habitat conversion and biodiversity loss [13], [14], [15], [16], [17]. However, as the human population increases, pressures on habitats are intensifying with unknown consequences for protected area effectiveness [18].

One of the regions at the forefront of global population and economic growth is South Asia. Comprising the countries of Bangladesh, Bhutan, India, Maldives, Nepal, Pakistan and Sri Lanka, South Asia's human population more than tripled between 1950 and 2009, from 473 million to 1.6 billion, and is projected to grow a further 41% by 2050 [19]. South Asian economies are mainly based on agriculture (for example, in Nepal over 90% of the population is involved in agriculture), meaning much of the region is now dominated by anthropogenic biomes [1]. Habitat degradation is occurring in all South Asian countries, mostly caused by intensive agricultural schemes, with much previously fallow land now being used for farming [20]. Consequently, areas of natural habitat and their associated biodiversity may have become increasingly restricted to protected areas.

The importance of protected areas in safeguarding biodiversity is now enshrined in Aichi Target 11 that forms part of the Strategic Plan for Biodiversity 2011–2020 of the Convention on Biological Diversity [21]. This Convention has been signed by 193 Parties, including all seven South Asian countries. Target 11 states that, by 2020, at least 17% of terrestrial and inland water habitat should be conserved effectively by protected areas or other similar area-based conservation measures. This is a very ambitious target and making significant progress towards it will require a close alignment of science, management and policy, and a sound understanding of the ecological integrity of established protected areas. Science has already demonstrated the contribution of protected areas to species coverage [22] and has developed methods for evaluating management and interventions [23]. However, targeted analyses of the extent to which particular protected area networks are meeting their biodiversity conservation objectives are in short supply given the number of protected areas in existence, though some studies are now emerging [24]. Surprisingly, given both the significant number of protected areas in the region (reflecting its global biodiversity importance) and its rapidly growing human population and economic power, assessments of the effectiveness of protected areas in South Asia are noticeably absent, particularly in comparison with the relative wealth of studies assessing protected areas in nearby Southeast Asia [18], [25], [26]. Within South Asia, some specific studies exist, such as those documenting lower rates of land cover change inside the Royal Chitwan National Park, Nepal [27] and the Tadoba Andhari Tiger Reserve, India [28] than outside these protected areas. There have been urgent calls for more studies in the South Asia region [29], [30], [31], particularly as the area has been identified a high priority for the addition of new protected areas [32]. Such assessments are also called for by the South Asia Environment Outlook, a consultative process with governments and other partners from South Asia, sub-regional intergovernmental agencies and experts [20].

Here we compare coverage by anthropogenic land uses inside and outside South Asia's protected area network, and measure the rates of habitat conversion over the last century both inside and outside the network. In so doing, we assess the extent to which the region's protected areas retain intact habitat, and provide insight into how protected areas across the planet might perform as human alteration of ecosystems continues to spread and intensify.

## Methods

We use a compare-to-everywhere approach in which we contrast (i) the extent of intact habitat inside protected areas with the entire unprotected landscape, and (ii) the historical trajectories of habitat clearance on protected and unprotected lands. This is in contrast with the often preferred method of using counterfactual data – see the discussion for an explanation of why we have taken a compare-to-everywhere approach here.

### (a) Mapping protected areas

**Figure 1:**
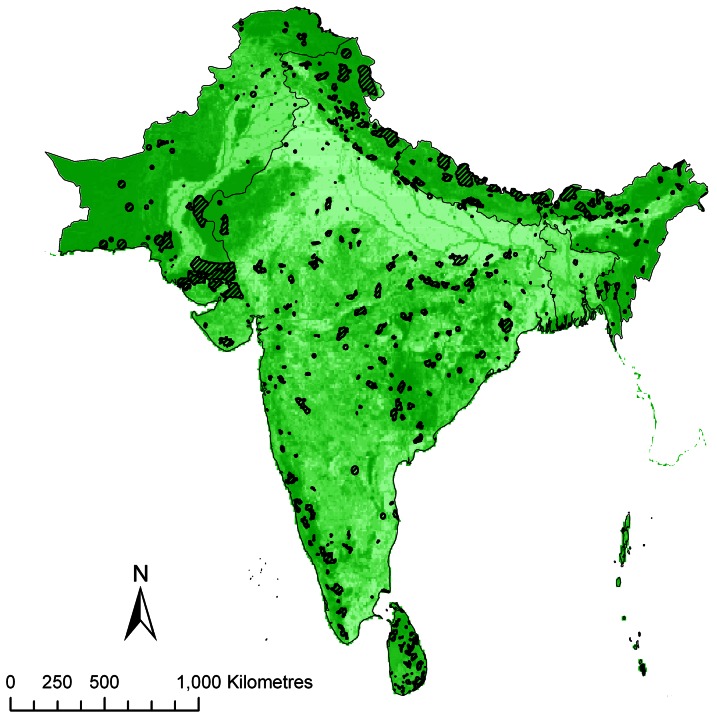
The spatial distribution of protected areas and habitat modification across South Asia. The green shading lightens with increasing levels of modification of natural habitat for agriculture in 2000 according to the HYDE 3.1 dataset [36].

Protected area shapefiles were downloaded from the 2009 World Database on Protected Areas [11]. We extracted the terrestrial areas in South Asia that were managed primarily for biodiversity conservation (IUCN management categories I–IV), in line with previous studies on protected area effectiveness [15], [33]. The Maldives does not have any terrestrial protected areas, only marine reserves, so does not contribute to this analysis. Protected areas were mapped as they existed in 2005 [11], allowing us to align the protected area boundaries with three independent land cover datasets [34], [35], [36] (see below). Being below the spatial resolution of the land cover datasets, protected areas <1 km^2^ were excluded from the analysis as were sites that were given by point locations only. Areas with no establishment date (n = 5) were included for analyses of the current levels of modified land (as their inclusion in the World Database of Protected Areas indicates their confirmed establishment), but were removed for analyses of historical levels of conversion. The resulting 593 protected areas were merged into a single layer with any overlapping areas assigned the IUCN management category of the more strictly managed area. Additionally, historical protected area layers were made in the same way for each decade from 1880 to 2000, with each layer comprising only those areas that had been established by the relevant date. For all analyses, all land outwith the protected area boundaries is classed as ‘outside’. See Fig. 1 for a map of the protected areas.

### (b) Mapping land cover

Three independently derived and broadly contemporaneous land cover datasets were used to provide independent estimates of the current extent of intact habitat both inside and outside the protected area estate. The data were (i) GlobCover, a 300 m resolution land cover dataset built using MERIS (Medium Resolution Imaging Spectrometer) data gathered by the Envistat satellite between mid-2005 and mid-2006 [34]; (ii) Global Land Cover 2000, a 1 km dataset based on SPOT 4 satellite data acquired between November 1999 and December 2000 [35]; and (iii) the History Database of the Global Environment (HYDE) 3.1, that combines satellite data with modelled data at ∼8 km spatial resolution [36]. Both GlobCover and Global Land Cover 2000 provide land cover data for just one point in time, whereas the HYDE dataset provides estimates of land cover across numerous time periods. The HYDE data were constructed by superimposing historic data regarding the locations of agricultural landscapes onto estimates of the natural distribution of vegetation communities prior to human transformation. Subsequent expansion of these cleared landscapes was modelled using historical human population density derived from a number of sources. Resulting estimates of current and pre-agricultural land cover agree well with those generated by other models and measurements [37], [38], [39]. Classification uncertainty of the HYDE 3.1 dataset is estimated at 5% in 2000, 10% in 1900, and 25% in 1800 [36], and there is no reason to suppose that classification errors within the protected area network differ from those in unprotected lands. Full details of the methodology can be found in Klein Goldewijk et al [36].

Land cover classifications used in GlobCover and Global Land Cover 2000 were grouped into those broadly indicative of either converted or intact habitat (see table S1 and table S2). HYDE data are supplied as percentage values reflecting the extent of land conversion for agriculture, and these percentages were used directly (Fig. 1).

We used the three land cover datasets to produce three individual estimates of the present percentage of habitat modified by humans both inside and outside the protected area network. For the HYDE land cover dataset, data from 2000 were used to provide the closest match in time with the Globcover and Global Land Cover 2000 data, which were obtained between the end of 1999 and mid-2006. Each land cover classification was overlain onto the protected area system, yielding estimates for the percentage of land that is human modified both inside and outside the protected network.

### (c) Historical sequence of habitat conversion

In addition to providing an estimate of current levels of the amount of land cover that has been modified by humans, we investigated the historical rate of land cover change across South Asia. Here, we assessed whether land within protected areas has been less vulnerable to conversion, and hence better protected, than all land outside the protected area boundaries.

To undertake this analysis we used the HYDE 3.1 dataset, which models land cover across South Asia for various historical points in time. The strong agreement between HYDE 3.1 and the other two land cover datasets for the contemporaneous estimates of habitat clearance derived above suggests we can confidently investigate historical patterns using the HYDE 3.1 data. We used the HYDE 3.1 dataset to produce a decadal time series of land cover maps for South Asia from 1850 to 2000. Each map consisted of ∼8 km grid cells, with an estimate for each grid cell of the percentage of land that has been anthropogenically transformed within that cell. Urbanised areas are excluded from these percentage estimates [36], leading to a conservative effect on our results i.e. in some areas more land will have been converted for human use than our estimate suggests.

Each decadal habitat map was overlain onto the protected area system as it existed in that decade, allowing us to calculate estimates for the percentage of land that had been anthropogenically transformed in each decadal time slice, inside and outside the protected area network. Therefore, our use of the term ‘habitat conversion’ does not refer solely to land use change post-gazettement but to that which has occurred at any point in time. We excluded five sites for which the establishment date was unknown and a further 27 sites gazetted after 1994, because insufficient time had elapsed to estimate clearance rates following gazettement.

## Results

### Current land cover distribution

Since 1950, South Asia's protected area system increased 64-fold [11], a significant legislative commitment in the face of rapid anthropogenic growth. As of 2005, between 24.0% and 27.9% of the habitat within the protected area estate was human modified, with very close agreement among the three independent sources of land cover data and no obvious impact of the spatial resolution of the land cover data on the estimates (Table 1). The mean amount of human modified land inside individual protected areas varied from 39.4% to 43.1% among the three data sets (Table 1). To investigate whether the minimum protected area size of 1 km^2^ affected these values unduly, we recalculated the figures including only protected areas larger than 5 km^2^, discovering that human modified habitat comprised 40.6%, 35.7% and 36.9% of the protected area network respectively for the GlobCover, GLC and HYDE estimates of land cover. Further restriction to a minimum size of 10 km^2^ yielded mean modified levels of 40.2%, 35.3% and 36.7%, and an even greater minimum threshold of 25 km^2^ gave values of 39.8%, 34.7% and 36.0%. This indicates a minimal effect of the minimum protected area size threshold on our results.

**Table 1:** Habitat conversion in South Asia.

Land cover dataset	Approximate spatial resolution, degrees (km)	Mean percent habitat conversion among protected areas	Total percent conversion inside protected area estate	Total percent conversion outside protected area estate
GlobCover [34]	1/360 (∼0.3)	43.1	27.86	59.59
GLC2000 [35]	1/112 (∼1)	40.3	23.98	53.75
HYDE 3.1 [36]	1/12 (∼8)	39.4	26.59	51.16

Values indicate the percentage of original natural habitat that had been converted to human use by 2000, as measured by three independent land cover datasets. In 2000, the 593 protected areas in South Asia covered 228,763 km^2^, or 5.5% of the region's land surface.

**Figure 2:**
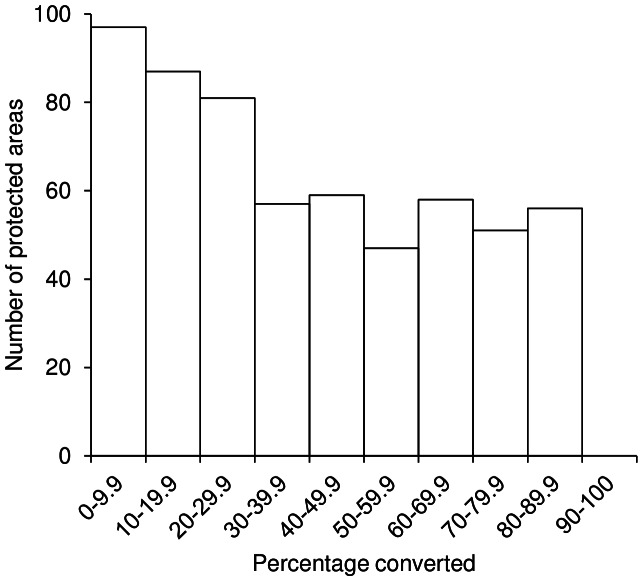
Frequency distribution of habitat conversion across South Asia's 593 protected areas. Using HYDE 3.1 land cover data for 2000 [36], the number of protected areas that have undergone each level of habitat conversion as measured at 10 percentage increments.

**Figure 3:**
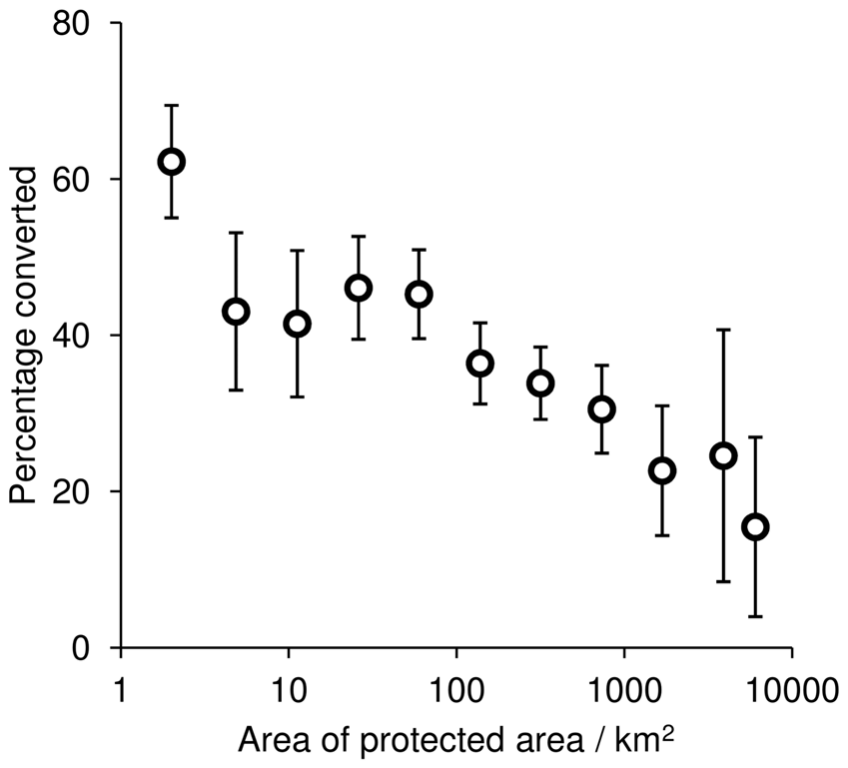
Proportional habitat conversion declines with increasing protected area size. The plot shows mean (±1 SE) percentage of habitat conversion across the full range of protected area sizes, grouped into 11 quantiles.

More detailed analysis with the HYDE 3.1 dataset showed that 212 of the 593 protected areas had more than half of their habitat modified for human use in 2000, with several sites displaying almost total habitat transformation (Fig. 2). The slope of the relationship between protected area size and the area of modified land within it was significantly below unity (standardised major axis regression: β = 0.7627, 95% CI = 0.7469–0.7790, *p*<0.001) indicating that, on average, larger protected areas have been subject to lower levels of habitat modification (Fig. 3). Overall, human modification of habitat was much more pervasive outside the protected area system than inside it, with estimates ranging between 51.2% and 59.6%, again with broad agreement among the three data sources (Table 1).

### Historical habitat conversion

**Figure 4:**
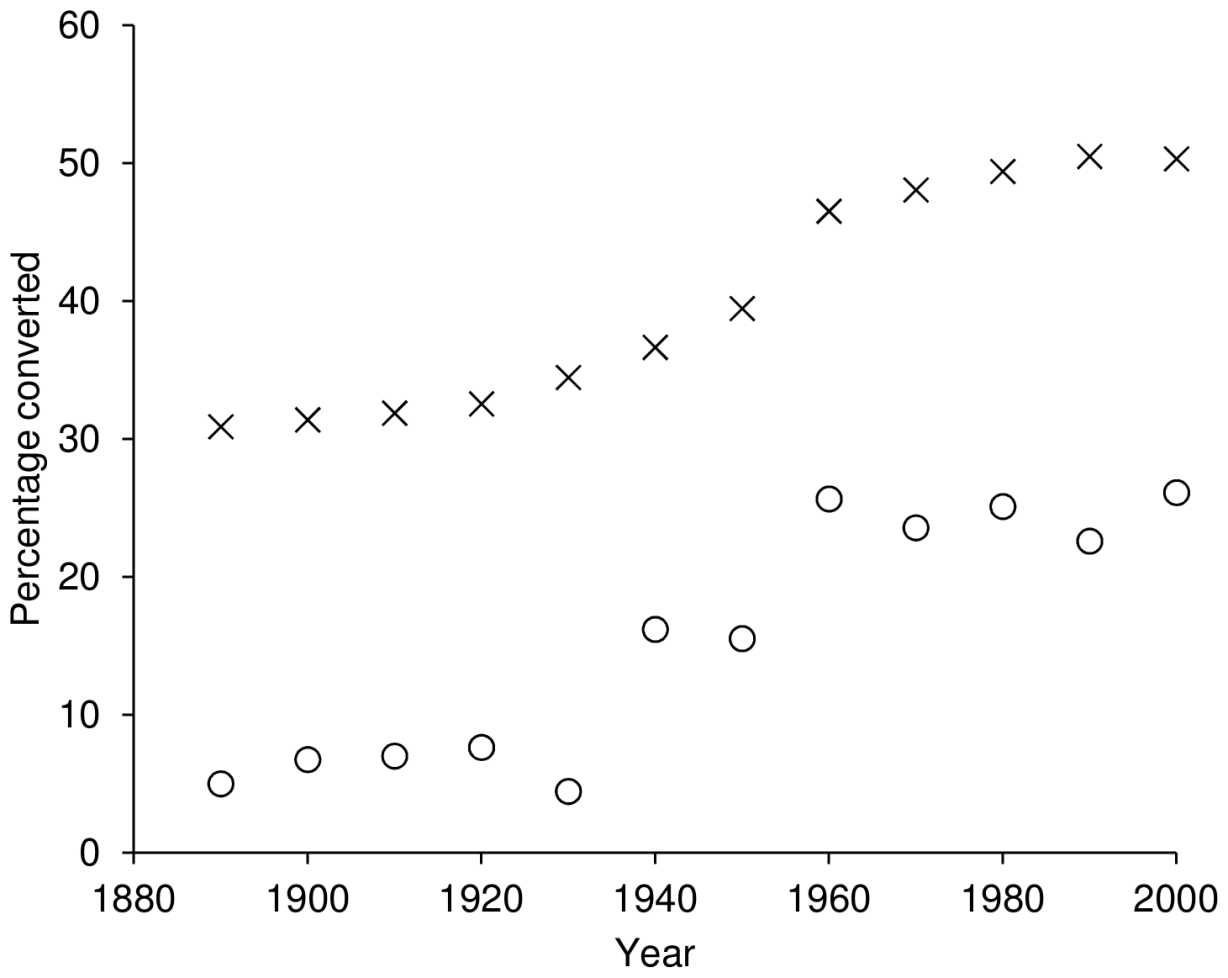
Habitat conversion inside (circles) and outside (crosses) South Asia's protected areas. Estimated using the HYDE 3.1 land cover dataset [36], the slopes of these trajectories of habitat conversion over time are not statistically distinguishable (see text).

The proportion of human modified habitat inside the South Asian protected area system increased steadily from about 5% at the time of establishment of the first site in 1889 to 26% by the year 2000 (Fig. 4). Although this is much lower than the equivalent values for unprotected lands, which were 30% modified in 1889 and 50% in 2000, the pattern of increase in habitat conversion inside and outside protected areas over time was similar (Fig. 4). We used standardised major axis regressions to investigate this statistically using time as the independent variable and proportion of the area that had been modified as the dependent variable. The slope of increase in habitat clearance outside protected areas was 0.2164 (95% CI = 0.1819–0.2576), and that inside protected areas was 0.2347 (95% CI = 0.1816–0.3032), with the difference in slope being non-significant (*p* = 0.588; Fig. 4). Moreover, using individual protected areas as units for analysis, there was little evidence that gazettement slowed the rate of habitat conversion within protected sites. Prior to gazettement, the land inside what were to become the protected area boundaries was being converted at a mean rate of 1.31% per decade. This subsequently dropped to 1.01% per decade after gazettement. We compared the rate of habitat clearance before and after gazettement across the protected areas using a paired t-test and, despite considerable power, this difference was not statistically significant (t = 0.46, df = 560, p = 0.645), suggesting that protected areas in South Asia are not significantly decreasing habitat loss compared with unprotected lands. Habitat conversion rates within protected areas appear to have stabilised somewhat since the 1960s but this at least partially reflects a general slowdown in the rate of conversion across the region at large (Fig. 4).

## Discussion

Our results indicate that habitat within South Asia's protected area system has been substantially modified and that this situation has been unfolding since many of the protected areas were originally gazetted. It is now at a stage where it merits urgent attention. Our most noteworthy results are that rates of clearance inside the protected area estate are not statistically distinguishable from those outside, and that rates of clearance inside protected areas do not significantly decrease after gazettement. This clearly raises doubts about South Asia's ability to contribute to the Convention on Biological Diversity's Aichi Target 11, which sets out both quantitative (17% of terrestrial and inland water habitats) and qualitative (effectively managed) criteria [21].

Around a quarter of the total land surface inside South Asia's protected area estate has been modified by anthropogenic uses, and as a result, protected areas might not be conserving biodiversity to the level that their extent might suggest. Each protected area has had, on average, more than a third of its land cleared for human use. Whilst land outside protected areas has been modified to a much greater degree, with more than half of the habitat being transformed for human use, the scale of conversion within protected areas is clearly a major concern. This is especially so if some protected areas have been sited in locations where land use change pressure is relatively low [15]. This suggests two things. Firstly, that the conversion of habitat in currently accessible areas that can be efficiently modified is higher, possibly substantially so given the presence of significant mountainous and arid ecosystems in the region. Secondly, with increasing pressure from both an increased population and a need for new land, these currently unattractive areas will also be converted from natural to modified habitats unless they are afforded effective protection and management.

Comparable results have been described from other parts of the world. Rayn and Sutherland [40] found similar rates of forest deforestation between land inside and outside protected areas in Mexico, and no difference in forest loss in protected land before and after gazettement. Similarly, Curran et al [18] report that more than 56% of protected lowland forests were degraded between 1985 and 2001 in Kalimantan (Indonesian Borneo). A recent study by Mora and Sale [41] suggests that the effectiveness of existing protected areas and the rate at which new protected areas are being established will not be sufficient to combat current biodiversity losses. All of this suggests that it is imperative that we change our way of thinking.

### Methodological robustness

Remote-sensing is one of the most widely used tools in large-scale analyses of habitat change [42], although the resulting data do not always capture fine scale degradation that does not completely transform the habitat [43]. Field-based studies would obviously improve our estimates of habitat conversion but are impractical for large-scale projects. Smaller-scale studies assessing the effectiveness of individual protected areas in South Asia have also found severe habitat degradation as a result of insufficient management [44], lending credence to the findings of our coarser-scale study.

The importance of using good counterfactuals when evaluating conservation interventions is widely acknowledged [45], [46], [47] and typically preferable in studies of protected area effectiveness [45]. The use of such counterfactual data can eliminate the false attribution of habitat intactness within protected areas to protection status alone, for example where protected area placement is biased toward high altitude, remote locations which have a lower probability of conversion [48]. In such cases, lower levels of habitat conversion may well be attributable to the location of the protected area rather than its protected status *per se*. Here we purposely use a ‘compare to everywhere’ approach [15]; a technique which has been criticised as, if protected areas are non-randomly distributed across a landscape on lands with lower deforestation probabilities, it may overestimate habitat protection within the system. However, our finding that there is no difference in clearance rates across the protection boundary is strengthened, not weakened, by our choice of method since, given that protected areas tend to be sited in areas where the probability of clearance is lower, our conclusion is conservative. We may in fact be overestimating the effectiveness of protected areas to conserve habitat in the region, in which case the crisis is even worse than we conclude. In a study of protected area effectiveness in 147 countries, Joppa and Pfaff [49] found that, on average, comparisons that control for differing land characteristics reduced estimated effectiveness by 50%. Our additional ‘compare to nearby time’ approach uses a more robust counterfactual comparing rates of conversion inside and outside the system before and after gazettement and confirms our findings that the protected area system of South Asia has not halted habitat conversion. Our study could be extended to compare deforestation rates between protected and unprotected lands with similar land characteristics.

### Size and location of protected areas

It has long been acknowledged that many of South Asia's protected areas may be too small to be viable and that they require careful management to provide conservation benefit in the long term [50]. Indeed, we find that it is the smaller protected areas that have proved particularly vulnerable, a result that is also found in other areas around the world [51]. The negative relationship between protected area size and levels of habitat conversion suggests one of two scenarios, or a combination of both. Firstly, smaller protected areas have, on average, a longer boundary relative to their area, which might increase vulnerability to clearance leaking contagiously across their borders. Additionally, they are often linked to larger scale ecosystems outside of their boundary and so are heavily influenced by neighbouring land cover change [52]. Secondly, larger protected areas tend to be sited in places that are less desirable for anthropogenic use and are less prone to habitat loss [49]. For example, Joppa and Pfaff [49] found that there is a bias towards siting protected areas in “rock and ice”, which is of limited use for establishing agriculture or urban infrastructure.

### Where do we go from here?

Our finding that the gazzetement and current management of protected areas has no significant impact on habitat modification suggests that the total area of land currently classed as protected in South Asia does not represent continuously intact natural habitat. Furthermore, it implies that the shelter afforded by the protected area system is not adequate to resist continued land clearance and, therefore, there is a pressing need to update management for these areas.

Most immediately, degazettement or realignment of protected area boundaries in the most irreparably degraded areas would more honestly reflect the contribution of these sites to the 2020 target of the Convention on Biological Diversity, whereby 17% of the world's terrestrial surface must be conserved through area-based measures [21]. Coupled with this, remotely sensed information on land cover integrity could be usefully deployed in this region to monitor changing vegetation status within protected area boundaries.

Retention of habitat is of course only one method of measuring the effectiveness of the protected area system. Other aspects of biodiversity will also be at risk through, for example, poaching, harvesting of non-timber forest products and grazing [17], [53], [54]. Whilst habitat cover is a useful proxy for protected area effectiveness there is an urgent need for research to assess effectiveness using species data. Basing the evaluation of protected area performance on specific conservation outcomes as advocated by Boitani et al [55] would aid in monitoring an area's practical effectiveness, specifically by tracking certain indicators, such as species richness, phylogenetic distinctiveness, vulnerability and irreplaceability.

Improved enforcement to abate habitat loss within protected areas is likely to prove difficult in South Asia, where rural population densities are high and many livelihoods rely on small-scale agriculture [50]. Programmes in India to resettle human communities living within protected areas often prove to be controversial, lengthy and expensive, and can lead to significant social unrest and long term negative impacts on traditional communities [53]. Indeed, a wave of degazettement of India's protected areas in the 1920s, and again more recently, indicates the difficulty of maintaining such sites under significant social and economic pressure [56]. However, there is evidence that suggests local residents may support conservation in surrounding protected areas if their livelihood needs are met [57], and that more relaxed management regimes can provide livelihood and biodiversity benefits [58].

Another potential solution is to undertake restoration activity that might help a degraded area recover its condition [59]. However, some protected areas are so heavily altered that it is unlikely they could return to a functioning condition through such means and, given the extent of habitat conversion in South Asia, the availability of replacement intact sites [24], [60] may be limited. Reforestation efforts are underway with forest cover increasing in Bangladesh, India and Pakistan in the last decade but plantations do not necessarily reflect original diversity nor provide the same environmental services [20]. Where formal protection of land outside the protected area estate is not possible, other conservation interventions could be considered. As well as being important for biodiversity in its own right, land outside protected area boundaries can be vital for achieving broader conservation objectives [61]. Without these larger connecting areas of habitat, species can become increasingly isolated within protected areas [62].

### Concluding remarks

Multiple factors have led to habitat conversion in South Asia; agricultural conversion, fuelwood and fodder extraction, logging, grazing, flooding and wildfire (both natural and anthropogenic) and tourism [31] (and references therein). Whilst our study highlights the urgent need for a review of protected area management, we have not yet discovered why the performance of individual protected areas differs so markedly. The identification of factors that have contributed to the success or failure of individual protected areas would require the extension of this study and the use of methods such as those developed by Joppa and Pfaff [15]. With regards to South Asia, particular attention should be paid to those regions regarded as high priority for protected area establishment, namely the Western Ghats, Sri Lanka and the eastern Himalayas [32]. Such research is increasingly urgent if we are to realise the full potential of protected areas in South Asia.

Protected areas have long been viewed as a cornerstone of biodiversity conservation across the planet and this role is recognised in a globally agreed target to be achieved by 2020. However, as we show here, protection is not an automatic consequence of protected area gazettement, with the result that much of South Asia's protected area estate contains modified habitats of unknown conservation value.

## Supporting Information

Table S1 **Land cover values in the GlobCover dataset** [**34**] **.** GlobCover values 11, 14, 20, 30 and 190 (using level 1 legend descriptions) were treated as converted habitat and all other values as primarily natural habitat.
(DOC)

Table S2 **Land cover values in the Global Land Cover 2000 dataset** [**35**] **.** Global Land Cover 2000 values 16–18 and 22 were treated as converted habitat and categories 1–15 and 19–21 were treated as primarily natural habitat.
(DOC)
