# Supplementary material for: Protected Areas in South Asia Have Not Prevented Habitat Loss: A Study Using Historical Models of Land-Use Change
Source: PLoS One. 2013 May 31;8(5):e65298. doi: 10.1371/journal.pone.0065298 (PMC3669372; doi:10.1371/journal.pone.0065298)
Supplement: Table S1 — Land cover values in the GlobCover dataset [34] . GlobCover values 11, 14, 20, 30 and 190 (using level 1 legend descriptions) were treated as converted habitat and all other values as primarily natural habitat. (DOC) [file pone.0065298.s001.doc]

**Table S1. Land cover values in the GlobCover dataset [34].** GlobCover values 11, 14, 20, 30 and 190 (using level 1 legend descriptions) were treated as converted habitat and all other values as primarily natural habitat.

| **Global GlobCover legend (level 1)** | **Global GlobCover legend (level 2)** | **Value** |
| --- | --- | --- |
| Post-flooding or irrigated croplands (or aquatic) |  | 11 |
|  | Post-flooding or irrigated shrub or tree crops | 12 |
|  | Post-flooding or irrigated herbaceous crops | 13 |
| Rainfed croplands |  | 14 |
|  | Rainfed herbaceous crops | 15 |
|  | Rainfed shrub or tree crops (cash crops, vineyards, olive tree, orchards...) | 16 |
| Mosaic cropland (50-70%) / vegetation (20-50%) |  | 20 |
|  | Mosaic cropland (50-70%) / grassland or shrubland (20-50%) | 21 |
|  | Mosaic cropland (50-70%) / forest (20-50%) | 22 |
| Mosaic vegetation (50-70%) / cropland (20-50%) |  | 30 |
|  | Mosaic grassland or shrubland (50-70%) / cropland (20-50%) | 31 |
|  | Mosaic forest (50-70%) / cropland (20-50%) | 32 |
| Closed to open (>15%) broadleaved evergreen or semi-deciduous forest (>5m) |  | 40 |
|  | Closed (>40%) broadleaved evergreen and/or semi-deciduous forest (>5m) | 41 |
|  | Open (15-40%) broadleaved semi-deciduous and/or evergreen forest with emergents (>5m) | 42 |
| Closed (>40%) broadleaved deciduous forest (>5m) |  | 50 |
| Open (15-40%) broadleaved deciduous forest/woodland (>5m) |  | 60 |
| Closed (>40%) needleleaved evergreen forest (>5m) |  | 70 |
| Open (15-40%) needeleaved deciduous or evergreen forest (>5m) |  | 90 |
|  | Open (15-40%) needleleaved deciduous forest (>5m) | 91 |
|  | Open (15-40%) needleleaved evergreen forest (>5m) | 92 |
| Closed to open (>15%) mixed broadleaved and needleleaved forest (>5m) |  | 100 |
|  |  |  |
| **Global GlobCover legend (level 1)** | **Global GlobCover legend (level 2)** | **Value** |
|  | Closed (>40%) mixed broadleaved and needleleaved forest (>5m) | 101 |
|  | Open (15-40%) mixed broadleaved and needleleaved forest (>5m) | 102 |
| Mosaic forest or shrubland (50-70%) / grassland (20-50%) |  | 110 |
| Mosaic grassland (50-70%) / forest or shrubland (20-50%) |  | 120 |
| Closed to open (>15%) (broadleaved or needleleaved, evergreen or deciduous) shrubland (<5m) |  | 130 |
|  | Closed to open (>15%) broadleaved or needleleaved evergreen shrubland (<5m) | 131 |
|  | Closed to open (>15%) broadleaved evergreen shrubland (<5m) | 132 |
|  | Closed to open (>15%) needleleaved evergreen shrubland (<5m) | 133 |
|  | Closed to open (>15%) broadleaved deciduous shrubland (<5m) | 134 |
|  | Closed (>40%) broadleaved deciduous shrubland (<5m) | 135 |
|  | Open (15-40%) broadleaved deciduous shrubland (<5m) | 136 |
| Closed to open (>15%) herbaceous vegetation (grassland, savannas or lichens/mosses) |  | 140 |
|  | Closed (>40%) grassland | 141 |
|  | Closed (>40%) grassland with sparse (<15%) trees or shrubs | 142 |
|  | Open (15-40%) grassland | 143 |
|  | Open (15-40%) grassland with sparse (<15%) trees or shrubs | 144 |
|  | Lichens or mosses | 145 |
| Sparse (<15%) vegetation |  | 150 |
|  | Sparse (<15%) grassland | 151 |
|  | Sparse (<15%) shrubland | 152 |
|  | Sparse (<15%) trees | 153 |
| Closed to open (>15%) broadleaved forest regularly flooded (semi-permanently or temporarily) – Fresh or brackish water |  | 160 |
|  | Closed to open broadleaved forest on (semi-) permanently flooded land – Fresh water | 161 |
|  | Closed to open broadleaved forest on temporarily flooded land – Fresh water | 162 |
| Closed (>40%) broadleaved forest or shrubland permanently flooded – Saline or brackish water |  | 170 |
| Closed to open (>15%) grassland or woody vegetation on regularly flooded or waterlogged soil – Fresh, brackish or saline water |  | 180 |
| **Global GlobCover legend (level 1)** | **Global GlobCover legend (level 2)** | **Value** |
|  | Closed to open (>15%) woody vegetation on regularly flooded or waterlogged soil – Fresh or brackish water | 181 |
|  | Closed to open (>15%) woody vegetation on temporarily flooded land | 182 |
|  | Closed to open (>15%) woody vegetation on permanently flooded land | 183 |
|  | Closed to open (>15%) woody vegetation on waterlogged soil | 184 |
|  | Closed to open (>15%) grassland on regularly flooded or waterlogged soil – Fresh or brackish water | 185 |
|  | Closed to open (>15%) grassland on temporarily flooded land | 186 |
|  | Closed to open (>15%) grassland on permanently flooded land | 187 |
|  | Closed to open (>15%) grassland on waterlogged soil | 188 |
| Artificial surfaces and associated areas (Urban areas >50%) |  | 190 |
| Bare areas |  | 200 |
|  | Consolidated bare areas (hardpans, gravels, bare rock, stones, boulders) | 201 |
|  | Non-consolidated bare areas (sandy desert) | 202 |
|  | Salt hardpans | 203 |
| Water bodies |  | 210 |
| Permanent snow and ice |  | 220 |
