# Supplementary material for: Protected Areas in South Asia Have Not Prevented Habitat Loss: A Study Using Historical Models of Land-Use Change
Source: PLoS One. 2013 May 31;8(5):e65298. doi: 10.1371/journal.pone.0065298 (PMC3669372; doi:10.1371/journal.pone.0065298)
Supplement: Table S2 — Land cover values in the Global Land Cover 2000 dataset [35] . Global Land Cover 2000 values 16–18 and 22 were treated as converted habitat and categories 1–15 and 19–21 were treated as primarily natural habitat. (DOC) [file pone.0065298.s002.doc]

**Table S2. Land cover values in the Global Land Cover 2000 dataset [35].** Global Land Cover 2000 values 16-18 and 22 were treated as converted habitat and categories 1-15 and 19-21 were treated as primarily natural habitat.

| **GLC Global Class** | **Value** |
| --- | --- |
| Tree cover, broadleaved, evergreen | 1 |
| Tree cover, broadleaved, deciduous, closed | 2 |
| Tree cover, broadleaved, deciduous, open | 3 |
| Tree cover, needle-leaved, evergreen | 4 |
| Tree cover, needle-leaved, deciduous | 5 |
| Tree cover, mixed leaf type | 6 |
| Tree cover, regularly flooded, fresh water (and brackish) | 7 |
| Tree cover, regularly flooded, saline water | 8 |
| Mosaic: tree cover/other natural vegetation | 9 |
| Tree cover, burnt | 10 |
| Shrub cover, closed-open, evergreen | 11 |
| Shrub cover, closed-open, deciduous | 12 |
| Herbaceous cover, closed-open | 13 |
| Sparse herbaceous or sparse shrub cover | 14 |
| Regularly flooded shrub and/or herbaceous cover | 15 |
| Cultivated and managed areas | 16 |
| Mosaic: cropland/tree cover/other natural vegetation | 17 |
| Mosaic: cropland/shrub or grass cover | 18 |
| Bare areas | 19 |
| Water bodies | 20 |
| Snow and ice | 21 |
| Artificial surfaces and associated areas | 22 |
